# A localized pallidal physiomarker in Meige syndrome

**DOI:** 10.3389/fneur.2023.1286634

**Published:** 2023-12-21

**Authors:** Bo Zhang, Hong Tian, Yanbing Yu, Xueke Zhen, Li Zhang, Yue Yuan, Liang Wang

**Affiliations:** ^1^Key Laboratory of Brain Science, Zunyi Medical University, Zunyi, China; ^2^Guizhou Key Laboratory of Anesthesia and Organ Protection, Zunyi Medical University, Zunyi, China; ^3^Department of Neurosurgery, China-Japan Friendship Hospital, Beijing, China; ^4^CAS Key Laboratory of Mental Health, Institute of Psychology, Beijing, China; ^5^Department of Psychology, University of Chinese Academy of Sciences, Beijing, China

**Keywords:** Meige syndrome, deep brain stimulation, globus pallidus internus, local field potentials, theta oscillations

## Abstract

**Objectives:**

Oscillatory patterns in local field potentials (LFPs) have been recognized as disease-specific physiomarkers, particularly in the context of Parkinson’s disease and cervical dystonia. This characteristic oscillatory feature is currently employed in adaptive deep brain stimulation (aDBS). However, for other types of dystonia, especially Meige syndrome, a distinct physiomarker of this nature is yet to be identified.

**Methods:**

Local field potentials were recorded during microelectrode-guided deep brain stimulation surgery from 28 patients with primary Meige syndrome. Before surgery, the severity of patients’ motor syndrome were assessed using the Burke-Fahn-Marsden Dystonia Rating Scale-Motor (BFMDRS-M). An instantaneous oscillation detection method was employed to identify true narrowband oscillations. Subsequently, a linear mixed effects model was utilized to examine the relationship between oscillatory activities (including power amplitude and burst duration) and symptom severity.

**Results:**

The focal peaks of “oscillatory activities” detected were predominantly concentrated in the narrow theta band (4–8 Hz), constituting 81.5% of the total detected oscillations in all recording sites near active DBS contacts in the globus pallidus internus (GPi). The linear mixed effects model revealed a positive correlation between the theta burst duration and the severity of preoperative motor impairment, but no correlation with postoperative motor scores. Additionally, there was no significant lateralization effect observed between the left and right GPi.

**Conclusion:**

Our findings suggest that the exaggerated narrowband theta activity (mainly the burst duration) in the GPi is predictive of dystonia symptom severity and may be used as a physiomarker for optimized DBS target during surgery and adaptive DBS for the treatment of Meige syndrome.

## Introduction

Meige syndrome is a rare type of dystonia characterized chiefly by dystonic spasms of the facial muscles (1). Early treatment methods include oral medication (e.g., anticholinergics, levodopa, and dopamine receptor blocker) and local injection of botulinum toxin type-A (2). However, some patients are drug-resistant after long-term and repeated usage (3). For such patients, deep brain stimulation (DBS) of the internal globus pallidus (GPi) has been established as a highly effective treatment, which may arise from a modulation of aberrant neural population activity in the basal ganglia through high-frequency stimulation (4).

Oscillatory patterns of pallidal local field potentials (LFPs) have been demonstrated in a disease-specific manner. Converging evidence points to pathologically increased beta (13–35 Hz) (5) and broad low frequency (4–12 Hz) (6) oscillations in Parkinson’s disease (PD) and cervical dystonia, respectively. These uncovered aberrant oscillations have paved the way for their applications as physiological biomarkers for target locations and adaptive DBS (6, 7).

However, previous studies investigating neural oscillation features for dystonia have involved patients with a mixture of dystonia symptom (8, 9). Consequently, the findings may be influenced by the possibility that different types of dystonia exhibit variations in oscillatory activity, contributing to the broad range of abnormal low frequency patterns detected in previous studies (6, 8–11). Focusing on patients with specific dystonia types can enhance our understanding of the underlying pathological mechanisms. However, such investigations have been primarily conducted in cervical dystonia (6). For other types of dystonia, especially Meige syndrome, a distinctive physiomarker remains elusive.

In this study, we characterized neural oscillations in the GPi for patients with Meige syndrome. By using a real-time oscillation detection method and avoiding the confounding effect of broadband power shifts, we detected focal peaks exceeding the background power threshold and revealed the predominant theta oscillations (4–8 Hz). Also, the burst duration was quantitatively measured. We found that theta durations were positively correlated with motor impairment before surgery. These results provide novel insights in the pathophysiology of Meige syndrome and theta oscillations have a potential application as a physiomarker for aDBS treatment.

## Methods

### Patient information

Patients were diagnosed with Meige syndrome by expert neurologists. All patients included in the study needed to meet the following inclusion criteria: (1) inadequate symptom relief after treatment with oral medication or local botulinum toxin type-A injections; (2) normal preoperative brain MRI examination; (3) absence of psychosis, dementia, psychiatric disorders, or a history of trauma; (4) no medical history of exposure to poisons; (5) electrophysiological signals recorded without obvious interference noise and with a duration longer than 7 s. A total of 28 patients with medically intractable Meige syndrome were enrolled, with an average age of 57.3 ± 8.0 years (Table 1). Before surgery, motor functions in patients were assessed using the Burke-Fahn-Marsden Dystonia Rating Scale-Motor (BFMDRS-M; Table 2).

**Table 1:** Demographic information of patients.

Gender, *n*	
Male	10
Female	18
Age, years	
Range	39–70
Mean ± SD	57.3 ± 8.0
Disease duration, years	
Range	1.5–7
Mean ± SD	4.2 ± 1.8
Electrode type, *n*	
Pins L301	6
Pins L302	22
Symptom type, *n*	
Blepharospasm	11
Oromandibular	0
Both	17

**Table 2:** BFMDRS-M total and sub-item scores (before and 2 years after surgery).

Preoperative BFMDRS-M Scores	
*Total*	15.0 ± 6.96
*Sub-item*	
Eye	7.21 ± 0.99
Mouth/Jaw	3.79 ± 3.14
Speech/Swallow	4.00 ± 4.55
Preoperative BFMDRS-M Scores	
*Total*	6.57 ± 5.04
*Sub-item*	
Eye	3.21 ± 1.57
Mouth/Jaw	1.64 ± 1.73
Speech/Swallow	1.71 ± 2.87
Improvement	
*Total*	56.0% ± 22.0%
*Sub-item*	
Eye	55.0% ± 22.0%
Mouth/Jaw	55.6% ± 29.6%
Speech/Swallow	61.3% ± 36.4%

Clinical improvement was calculated by the formula: [Preoperative BFMDRS-M score – Postoperative BFMDRS-M score]/Preoperative BFMDRS-M score × 100%. The BFMDRS-M sub-score for neck was not listed here as no patients had neck dystonia symptoms.

### Operation procedure and patient follow-up

Detailed surgical procedures can be found in previous descriptions (12). Briefly, a high resolution 3 T MRI of each patient’s head was obtained a few days before surgery. On the day of surgery, a thin layer CT scan (spacing 1 mm) was conducted with a CRW™ stereotactic frame (Radionics Industries, Inc., United States) mounted on the patient’s head. Images from MRI and CT scans were further merged to determine both implant location and trajectory in bilateral GPi.

Electrophysiological signals were obtained during microelectrode recording-guided DBS surgery (13), and local field potentials were recorded with a sample rate of 760 Hz using NeuroNav™ (Alpha Omega Engineering, Israel). Throughout the entire recording process, patients remained awake, in a resting state, and under local anesthesia. Microelectrode recordings typically commenced 10 mm above the planned target point and advanced with a step of 0.5 mm. Once the target location was confirmed, the microelectrode was withdrawn, and a DBS electrode (Pins L301/L302, Beijing PINS Medical Co., Ltd., China) was implanted using the same trajectory. In some cases, multiple microelectrode trajectories may be explored, but our analysis considered only the microelectrode trajectory used for the final implantation of the DBS electrode. A thin layer CT scan of the head was conducted after surgery to verify the DBS electrode position. Patients were followed up to ensure no adverse side effects occurred, and patients’ BFMDRS-M scores were re-evaluated 2 years after surgery and compared with those assessed before surgery (Table 2).

### Data processing and analysis

Locations of DBS electrode contacts were reconstructed in the individual native space and converted into the standard Montreal Neurological Institute (MNI) space using Lead-DBS software (14). Subsequently, the locations of microelectrode recording sites in native space were calculated based on the locations of DBS electrodes implanted along the same trajectory and normalized to the standard MNI space using methods from previous study (15). Microelectrode recording sites within the GPi were then extracted within 2 mm of active DBS contacts. Each microelectrode recording site was considered only once, even if sometimes it fell within the 2 mm threshold of more than one active DBS contact. This radius was determined by the volume of activate tissue model proposed by Madler & Coenen (16), with parameters (k_1_ = −1.0473, k_2_ = 0.2786, k_3_ = 0.0009856), and average impedance (Ω = 2000) and stimulation voltage (U = 3.0 V) across all patients:


$$r\left(\Omega ,U\right)=-\frac{k_{3}\Omega -\sqrt{k_{3}^{2}\Omega^{2}+2k_{1}k_{3}\Omega +k_{1}^{2}+4k_{2}U}+k_{1}}{2k_{2}}$$

Local field potentials signals were initially filtered using a fourth order Bessel filter with a passband of 0.5–45 Hz that was below the 50 Hz power noise. The recording duration for each site was commonly set at 10 s, with a 7 s threshold used to exclude signals with excessively short recording times. The average recording duration included in the analysis was 9.9 ± 0.8 s. Signal quality was assessed through meticulous visual inspection, and only recorded sites free of obviously electrical noises were retained (a total of 404 recording sites from 28 patients). For signals from each recording site, we employed an oscillation detection method called the extended Better Oscillation Detection Toolbox (eBOSC) (17) to identify true oscillations and mitigate the confounding effects of broadband power shifts. In brief, a six-cycle Morlet wavelet with 33 logarithmically-spaced center frequencies ranging from 2 to 32 Hz was used to conduct time-frequency analysis of signals in each recording site (18). After the wavelet transform, 1 s at each segment’s borders were removed (i.e., 2 s in total) to exclude edge artifacts. Subsequently, the background power threshold was estimated based on the 95th percentile of a χ^2^(2)-distribution of power values. Finally, the wavelet-derived signals at a particular frequency and time window were regarded as true rhythms when and if when the power exceeded the predefined background power threshold and lasted for a minimum duration of three cycles. In the end, two characteristics of recorded signals, i.e., power amplitude and its duration (i.e., burst duration), were calculated. The power amplitude of the detected frequency band was normalized to the background power. By this means, both normalized power and burst duration of the specific frequency band can be well assessed.

The correlation between BFMDRS-M and one of two characteristics (the normalized power and duration of oscillation activity) were calculated using a linear mixed effect model (LME), which extended the simple linear model to allow both fixed and random effects considered together, and was widely used for non-independent data (19). Since each patient had several recorded sites, the LME equation was constructed in Matlab as:


$${}^{"}y\sim 1+x+{\left(1|\mathrm{Patient}\right)}^{"}$$

Where y denotes to the normalized power or duration of theta oscillations from all recorded sites of all patients, x denotes to the BFMDRS-M as a fixed-effect variable, patient is a random-effect variable. All data were reported as mean ± standard deviation (SD) and the significance level was set at 0.05.

## Results

### DBS electrode locations and stimulation parameters

**Figure 1:**
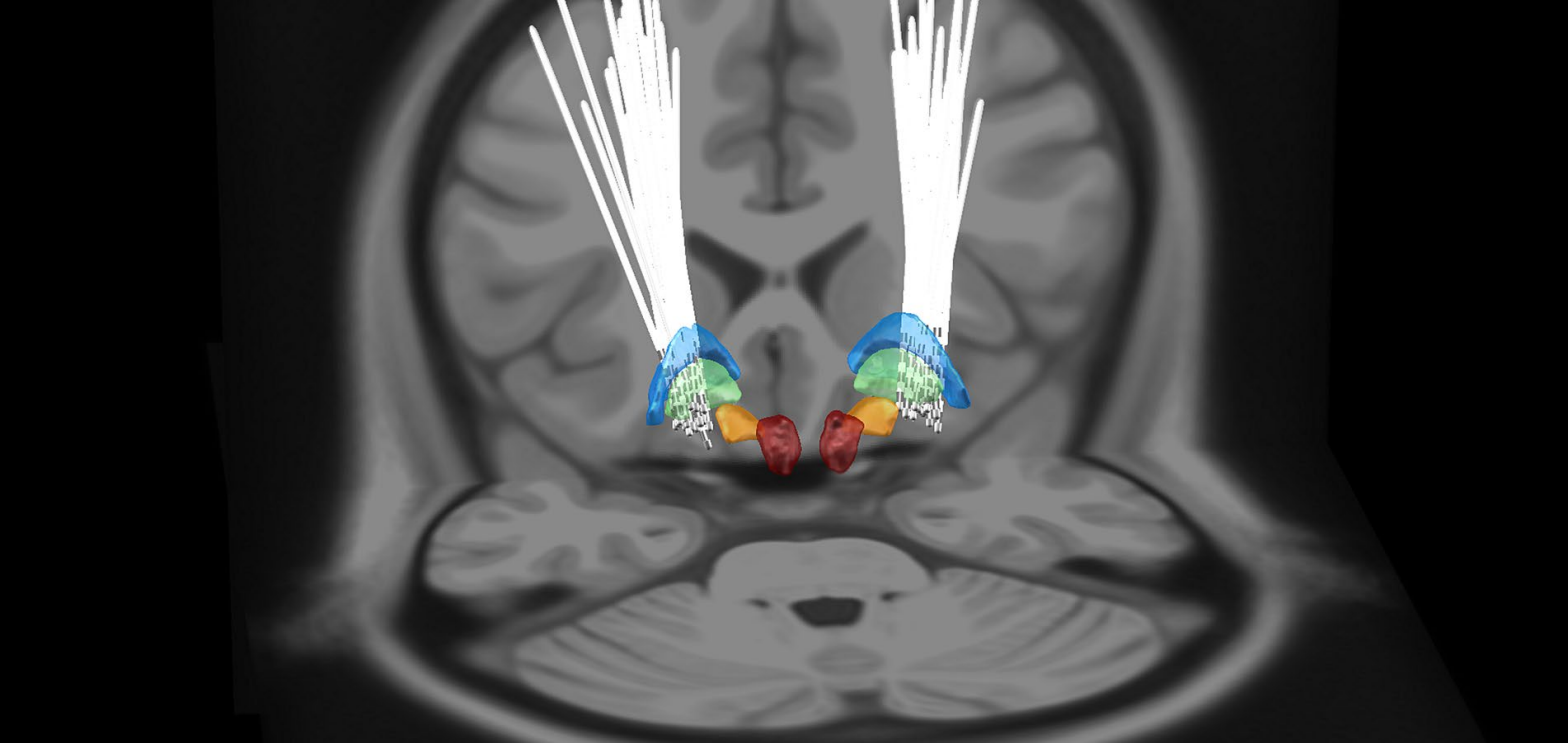
3D reconstruction of DBS leads for all patients in MNI space using Lead-DBS software. GPe, globus pallidus externus (blue); GPi, globus pallidus internus (green); STN, Subthalamic nucleus (orange); and RN, Red nucleus (red).

Deep brain stimulation electrodes were reconstructed using Lead-DBS (Figure 1). The monopolar stimulation mode was programmed for all patients on DBS leads, with the following stimulation parameters: a voltage of 3.0 ± 0.3 V; pulse width of 92.5 ± 15.5 μS; frequency of 149.5 ± 66.3 Hz. Coordinates of active contacts on DBS leads in the MNI space (left side: *x* = −20.2 ± 1.4 mm; *y* = −5.8 ± 2.1 mm; *z* = −1.5 ± 3.3 mm; right side: *x* = 21.5 ± 1.8 mm; *y* = −5.2 ± 2.4 mm; *z* = −1.8 ± 3.2 mm) were predominantly located in the posterior GPi.

### Neural oscillations at active DBS contacts

**Figure 2:**
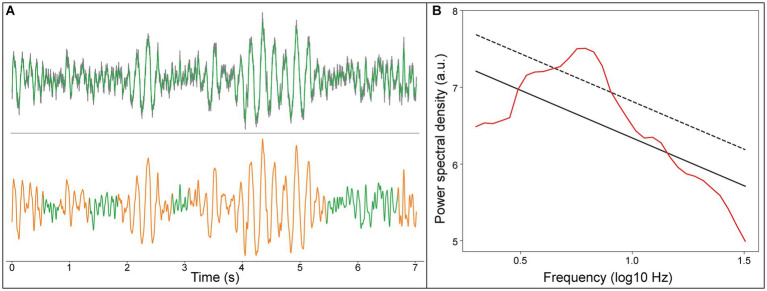
An example LFP signal of the recording site within 2 mm range of active DBS contacts in the GPi. **(A)** Upper panel, raw LFP signal (gray line), LFP signal filtered from 0.5 to 45 Hz (green line); Lower panel, the detected theta oscillation (orange line). **(B)** Rhythms of the LFP signal detected using the eBOSC algorithm, aperiodic components of the signal (black solid line), the estimated background power threshold (black dash line), and power spectrum of the signal (red line).

**Figure 3:**
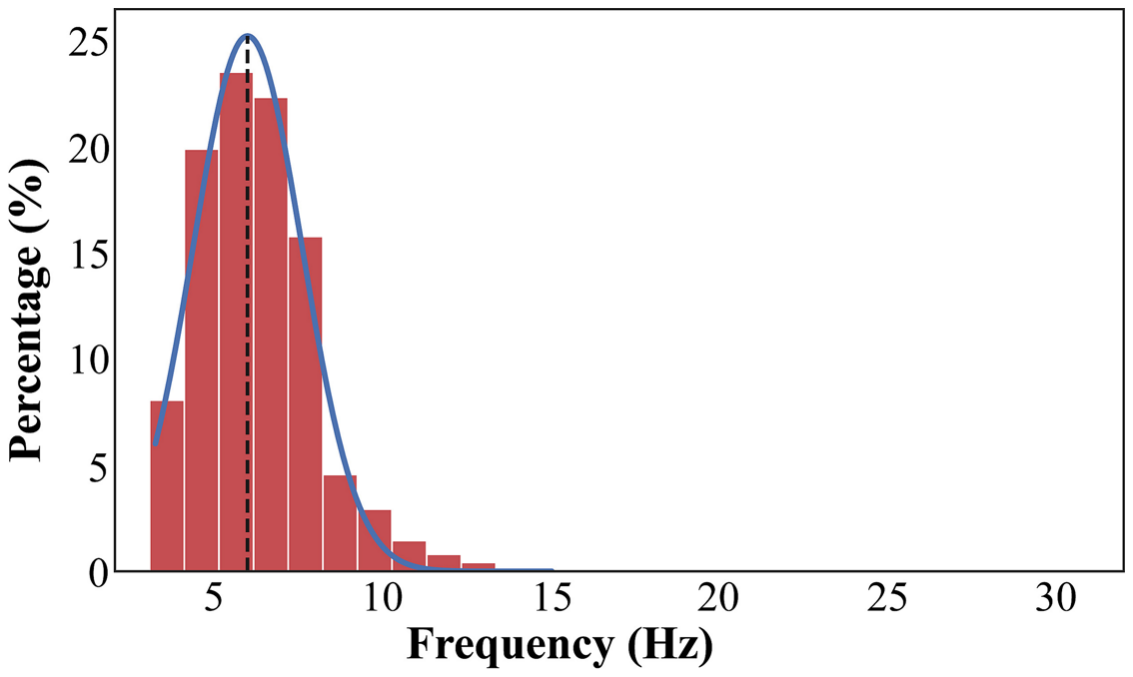
The distribution of frequencies that exceeds the background power threshold. The distribution of frequencies is fitted with a Gaussian kernel (blue line). The true oscillations are concentrated in 4–8 Hz, accounting for 81.5% of total detected oscillations in all recording sites within 2 mm range of active DBS contacts in the GPi. The overall peak frequency is about 6 Hz (black dash line).

We detected focal peak frequencies of narrowband oscillations extracted from the recorded signals using the eBOSC method (Figure 2). The histogram showed that the predominant peak of true oscillations concentrated within the frequency range of 4–8 Hz (Figure 3), accounting for 81.5% of the total detected oscillations in all recording sites within 2 mm of active DBS contacts in the GPi. Further, we fitted this distribution with a Gaussian kernel and identified that the peak frequency to be around 6 Hz (Figure 3). We also provided an averaged power spectral density (PSD) of signals from recording sites within a 2 mm range of active DBS contacts in the GPi (Supplementary Figure 1).

### Relationship between preoperative motor impairment and theta amplitude

The normalized power was utilized to assess its relationship with motor impairment severity, as it was less dependent on contact impedances and compatible across patients. The theta power was normalized to the background power at each recording site. We examined the relationship between motor impairment severity (assessed by preoperative BFMDRS-M) and the normalized theta power using the LME model. For recording sites within a 2 mm range of active DBS contacts in GPi, LME revealed a non-significantly correlation between motor impairment severity before surgery and the normalized theta power [*t*(402) = 1.31, *p* = 0.19]. For recording sites outside the 2 mm range of active DBS contacts in the GPi, the relationship was not significant [*t*(382) = 0.23, *p* = 0.82]. We also analyzed the lateralization of the relationship across the left and right GPi, but no significant difference appeared [*F*(1,401) = 0.40, *p* = 0.53].

### Relationship between preoperative motor impairment and theta duration

**Figure 4:**
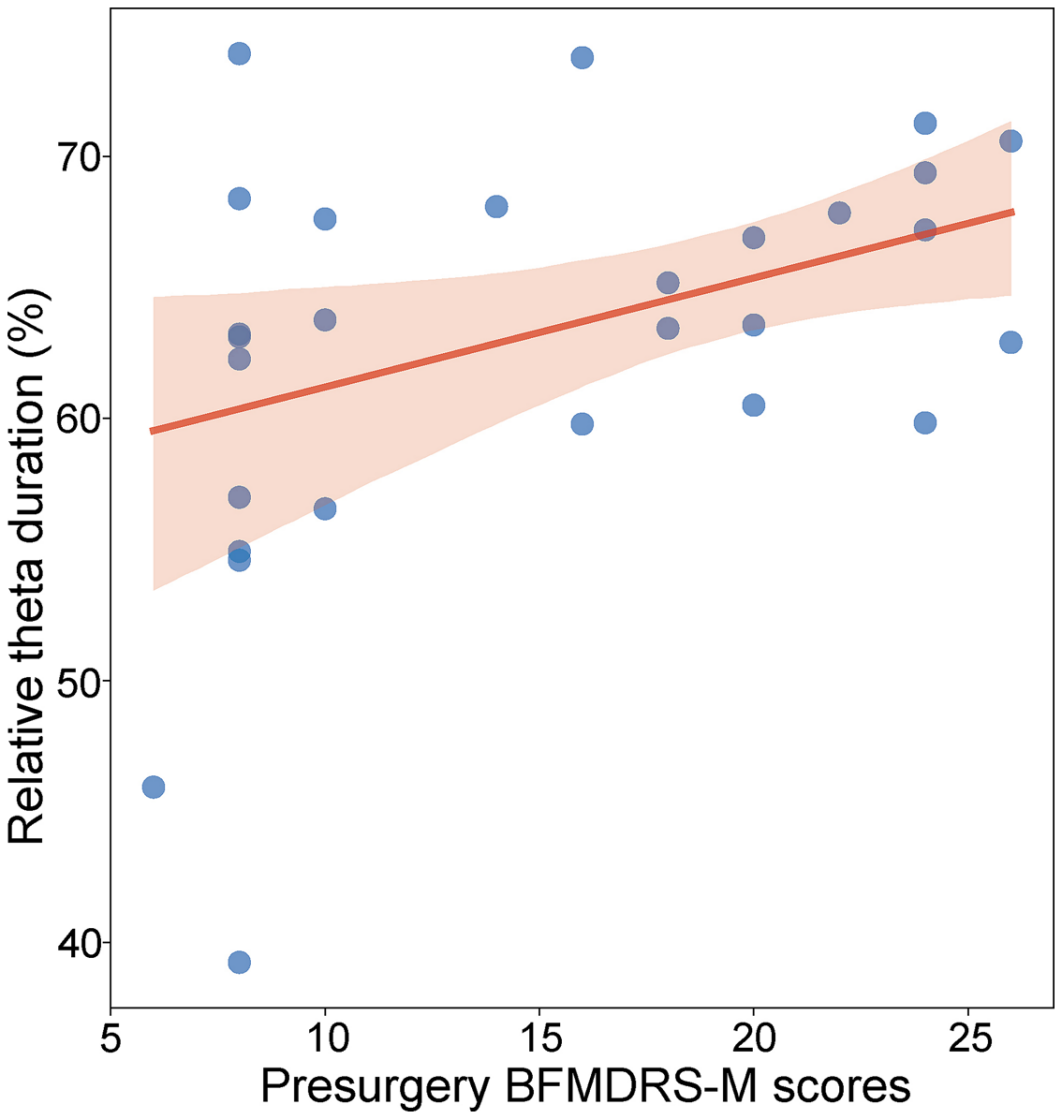
Relationship between preoperative BFMDRS-M scores and normalized theta duration (*p* = 0.030, *R*^2^ = 0.38, LME). Each blue dot represents the fixed effect of a patient. The shadow represents the 95% confidence interval for the robust-fit regression estimate.

Similar to the normalized theta power, theta burst duration was defined as the percentage of detected theta duration from the whole analyzed period. We examined the relationship between motor impairment severity before surgery and theta burst duration using the LME model. For recording sites within a 2 mm range of active DBS contacts in GPi, a significant positive relationship between motor impairment severity before surgery and the normalized theta duration was found [*t*(402) = 2.17, *p* = 0.030, *R*^2^ = 0.38, Figure 4]. For recording sites outside the 2 mm range of active DBS contacts in the GPi, the relationship was not significant [*t*(382) = 0.49, *p* = 0.62]. We also analyzed the lateralization of the relationship across the left and right GPi, but no significant difference appeared [*F*(1, 401) = 0.99; *p* = 0.32].

### Relationship between postoperative motor impairment, clinical improvement, and theta activity

The LME model was employed to assess the relationship between motor impairment severity two-years after surgery and theta activity (including power and duration), but the relationship was not found to be significant [*t*(402) = 0.42, *p* = 0.67; *t*(402) = 1.70, *p* = 0.10]. We also examined the relationship between clinical improvements 2-year after surgery and theta activity (including power and duration) using the LME model, but no significant correlation was observed [*t*(402) = 0.09, *p* = 0.93; *t*(402) = −0.92, *p* = 0.36].

## Discussion

In this study, we demonstrated that patients with Meige syndrome showed abnormally exaggerated theta (4–8 Hz) oscillations in the GPi near the active contacts. Theta burst durations were positively correlated with pre-operative motor symptom severity, but not correlated with post-operative motor score and clinical improvement. These results may help better understand the underlying pathological mechanisms of Meige syndrome and provide potential physiomarkers for aDBS.

### The advantages of this research

This study employed an oscillatory detection method to identify instantaneous neural oscillations, including amplitude and duration properties. In comparison to traditional approaches (e.g., fast Fourier transform and wavelet analysis), eBOSC does work well in instantaneously identifying the period with rhythmic components, allowing for precise characterization of electrophysiological signals obtained during brain surgery.

In addition, a linear mixed effect model was employed to assess the relationships between theta oscillations (i.e., amplitude and duration) and preoperative motor impairment severity. For data with repeated measures from the same subjects, such as in this study, Pearson correlations are not appropriate due to the violation of the sample independence. LME model extends traditionally linear regression models by combining both fixed and random effects together, making it increasingly popular in the fields, including medical research, social research, and neurophysiological research (19).

Finally, the most important and novel finding in this study was the association between the duration of theta oscillations and motor impairment severity before surgery, a relationship not demonstrated in previous studies of various subtypes of dystonia. Furthermore, theta oscillation duration may be more critical than the theta power index, as the results indicated in this study. Unlike oscillation amplitude, which reflects the size of neural population involved, oscillation duration reflects how long neural population are synchronized (17, 20). In PD, the extended duration of beta bursts in the subthalamus nucleus (STN) was presumed to increase the likelihood of phase coupling within local region as well as between hemispheres, leading to interrupted motor commands and subsequent motor impairment (5). STN-DBS has been suggested to trim long duration of beta bursts to achieve therapeutic effects in PD. (21) Although there is still much to learn about the pathophysiology of Meige syndrome and the underlying mechanisms of DBS therapy, the findings in this study undoubtedly contribute to advancing our understanding of these processes.

### Implications for adaptive DBS in primary Meige syndrome

Although conventional DBS (cDBS) in the GPi or STN has been proven to be a viable alternative for medically refractory Meige syndrome (4, 22) with an improvement range of 53–86% (23), its drawbacks should not be ignored. The programming for cDBS in primary Meige syndrome is time-consuming, involving a trial-and-error approach based on clinical responses, and usually takes weeks or months before a clinical change is observed. Additionally, significant adverse effects (e.g., dysarthria and parkinsonism) can be induced by cDBS in some patients, potentially increasing treatment costs and significantly affecting the patients’ quality of life.

One possible reason for side effects is that cDBS may interface with both pathological and physiological neural activities in the cortico-basal ganglia-thalamo-cortical (CBGTC) circuits (7, 24). It is possible that only disrupting pathological activities may help reduce these side effects, whereby pathological activities can be used as feedback signals to initiate DBS. This type of adaptive DBS has already been used for PD, whose elevated beta (13–30 Hz) activity (amplitude and duration) has been proven to be a disease-specific characteristic. For Meige syndrome, such biomarker has been missing to date.

This study investigated the neural oscillatory characteristics of the GPi in a relatively large group of patients with Meige syndrome. The exaggerated oscillation activity mainly concentrated on the theta band (4–8 Hz), and its burst duration was positively correlated with preoperative motor symptom severity. It is reasonable to infer that increased theta activities (4–8 Hz) might be a physiological characteristic for Meige syndrome, which is consistent with previous studies: (1) abnormally exaggerated low-frequency oscillations have been indicated in various types of dystonia (6, 8–11); (2) the longer duration of disease-related oscillation can lead to strong phasic synchronization in basal ganglia circuits and further affect normal motor functions, which has already been well investigated in PD. (5) Therefore, abnormal theta duration might be used to guide the design of aDBS for Meige syndrome in the future.

However, unlike the study on cervical dystonia by Neumann et al. (6), we did not observe a significant positive relationship between theta activities and syndrome improvement after surgery. We believe there are at least two possible reasons: (1) for Meige syndrome, both the assessment of symptom severity and the mechanisms underlying the therapeutic effects of DBS may be different from those in cervical dystonia; (2) the position of active DBS contacts and stimulation parameters were crucial factors affecting therapeutic effects. It is possible that these parameters were in their second-best states, as the adjustment of DBS parameters was difficult and time-consuming for Meige syndrome. Our finding that a positive relationship between theta activities and preoperative motor symptom severity only existed for recording sites within 2 mm near active DBS contacts may suggest this suboptimal parameter setting. Furthermore, an extended recording duration, an increased number of recording sites, and a larger sample size could potentially enhance our understanding of the intricate relationship between neural oscillations and treatment outcomes in Meige syndrome.

### Limitations

This study focused solely on investigating neural oscillations in the GPi of patients with Meige syndrome. However, the underlying pathology of Meige syndrome may involve the entire CBGTC circuits, given the close links in movement modulation between these areas. Both PD and dystonia have been proposed to be a network disorder (25, 26). The positive correlation between the duration of theta oscillation and symptom severity implied that increased synchrony within the CBGTC network may contribute to the pathophysiology of Meige syndrome, as well as its potential application for DBS treatment. However, this speculation needs to be tested in further studies. For example, by exploring: (1) characteristics of neural oscillations in other brain regions of the CBGTC network recorded for the same patient with Meige syndrome; (2) frequency-specific neural synchronization between different brain regions within the CBGTC network. Additionally, the development of an implantable neural interface for wirelessly recording neural signals (27) enables the probing of neural oscillations in target regions during both DBS ON and OFF states after surgery, while simultaneously recording symptom characteristics (e.g., electromyography). This approach allows for a more comprehensive characterization of neural oscillations during the onset of motor syndrome, contributing to a deeper understanding of the pathological mechanisms of Meige syndrome.

## Conclusion

This study found that patients with primary Meige syndrome exhibited exaggerated theta bursts in the GPi. Furthermore, the duration of theta activities near active contacts in the GPi showed a positive correlation with preoperative motor severity assessed by BFMDRS-M. Therefore, the elevated theta activity in the GPi may provide a clinically valuable biomarker for optimized DBS target and adaptive DBS in the treatment of primary Meige syndrome.

## Data availability statement

The raw data supporting the conclusions of this article will be made available by the authors, without undue reservation.

## Ethics statement

The studies involving humans were approved by the Ethics committee of China-Japan Friendship Hospital (2020-129-K82). The studies were conducted in accordance with the local legislation and institutional requirements. The participants provided their written informed consent to participate in this study.

## Author contributions

BZ: Conceptualization, Formal Analysis, Investigation, Methodology, Software, Visualization, Writing – original draft, Writing – review & editing. HT: Conceptualization, Data curation, Methodology, Writing – review & editing. YaY: Data curation, Methodology, Writing – review & editing. XZ: Data curation, Methodology, Writing – review & editing. LZ: Data curation, Methodology, Writing – review & editing. YuY: Data curation, Methodology, Writing – review & editing. LW: Conceptualization, Formal Analysis, Investigation, Methodology, Project administration, Software, Supervision, Visualization, Writing – original draft, Writing – review & editing.
