# Supplementary figures and images for: A localized pallidal physiomarker in Meige syndrome
Source: Front Neurol. 2023 Dec 21;14:1286634. doi: 10.3389/fneur.2023.1286634 (PMC10764606; doi:10.3389/fneur.2023.1286634)

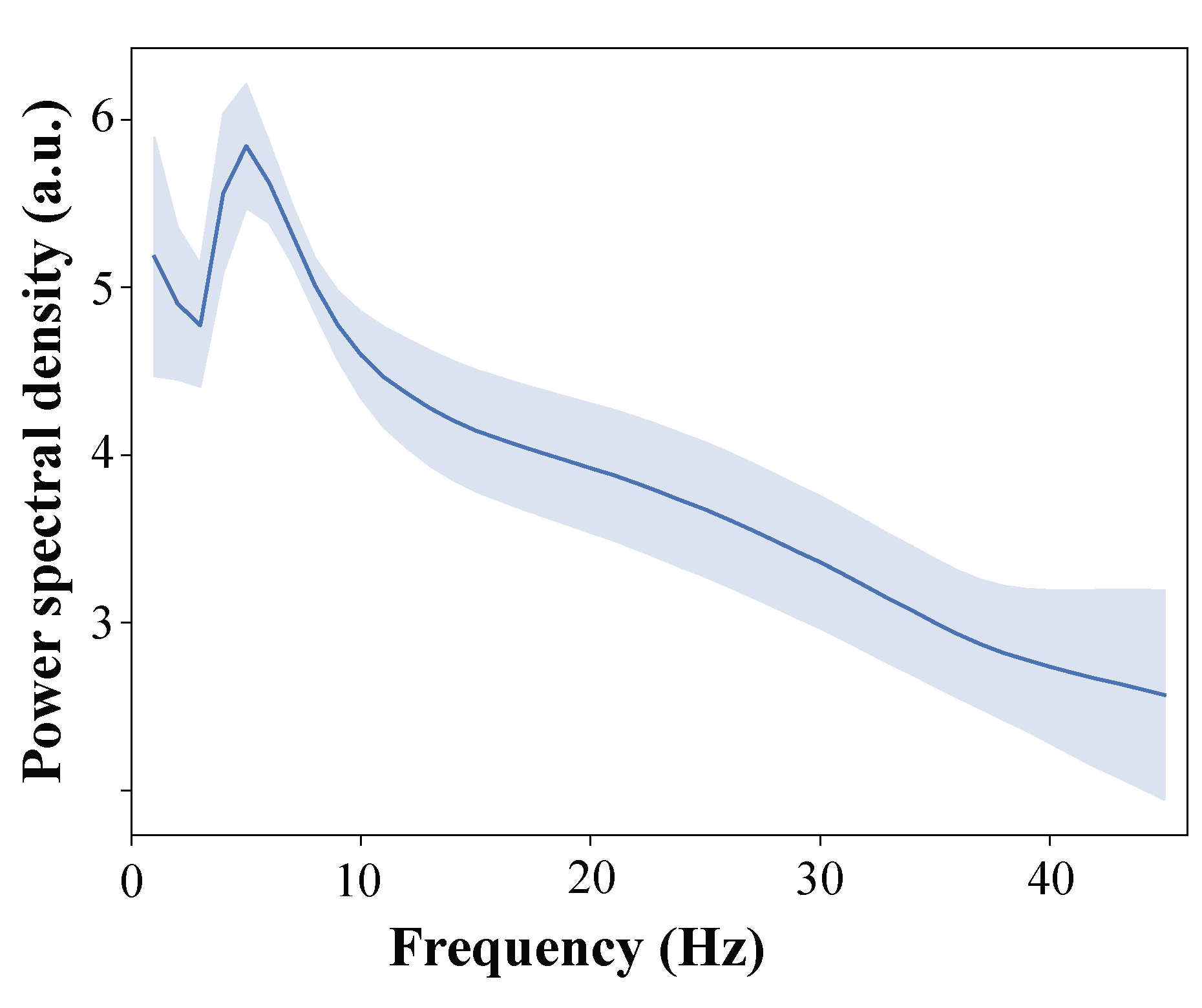

Supplement: Supplementary file 1 [file Image_1.JPEG]
